# High-Resolution Quantitative Reconstruction of Microvascular Architectures in Mouse Hepatocellular Carcinoma Models

**DOI:** 10.3390/cancers17162653

**Published:** 2025-08-14

**Authors:** Yan Zhao, Haogang Zhao, Xin Wang, Wei Dai, Xuhua Ren, Jing Wang, Guohong Cai

**Affiliations:** 1 Department of Liver Diseases and Interventional Radiology, Xi’an International Medical Center Hospital, Northwest University, Xi’an 710100, China; yanzhao211@163.com (Y.Z.); wangxin3@upenn.edu (X.W.); daiweisxhz@126.com (W.D.); 15793091216@163.com (X.R.); 2 State Key Laboratory for Manufacturing Systems Engineering, Xi’an 710054, China; 3 Department of Chemical Engineering, State Key Laboratory of Chemical Engineering, Tsinghua University, Beijing 100084, China; zhg23@mails.tsinghua.edu.cn; 4 Department of Nuclear Medicine, Xijing Hospital, Fourth Military Medical University, Xi’an 710032, China; 13909245902@163.com

**Keywords:** fluorescein angiography, Akt/Ras, HD-fMOST technique, liver vasculature, vascular imaging

## Abstract

**Background/Objectives**: Alterations in liver vascularization play a remarkable role in liver disease development, including hepatocellular carcinoma (HCC), but remain understudied. This study evaluated the hepatic microvascular imaging method and provided high-resolution quantitative anatomical data on the characteristics and architecture of liver vasculature in wild-type (WT) mice and HCC mouse models. **Methods**: C57BL/6 mice were injected with Akt/Ras or Sleeping Beauty transposon to induce HCC. Liver tissues from normal and Akt/Ras mice underwent hematoxylin and eosin, Masson’s trichrome, Ki67, and lymphatic endothelial receptor-1 staining. Using cutting-edge high-definition fluorescence micro-optical sectioning tomography, high-precision microvascular visualization of the liver was performed in WT and Akt/Ras HCC mice. **Results**: The sectioned volumes of normal and HCC liver tissues were 204.8 mm^3^ and 212.8 mm^3^, respectively. The microvascular systems associated with the tissues of the Akt/Ras HCC mouse were twisted, disordered, and compressed by tumor nodules. In the four tumor nodules, the path of the hepatic artery was more around the tumor edge, whereas the portal vein occupied the central position and constituted the main blood vessel entering the tumors. The porosity of HCC and paracancerous cirrhotic tissues was significantly less than that of normal tissues. The radii of the central vessels in the hepatic sinusoid of paratumoral cirrhotic tissues were significantly higher than those of normal tissues; however, the hepatic sinusoid density of paratumoral cirrhotic tissues was lower. **Conclusions**: This research provides a deeper understanding of the normal liver microvasculature and alterations in cases of cirrhosis and HCC, which complements scientific insights into liver morphology and physiology. This straightforward research approach involving the novel 3D liver microvasculature can be used in multiscale physiological and pathophysiological studies regarding liver diseases.

## Simple Summary

This study utilized the advanced HD-fMOST imaging technique to analyze 3D microvascular structures in normal and Akt/Ras-induced HCC mouse livers. The results revealed disordered, compressed vessels in HCC tissues, with portal veins (PVs)—not hepatic arteries (HAs)—serving as the primary blood supply for diffuse tumors. Additionally, sinusoidal networks were disrupted in cirrhotic and tumor tissues, showing reduced density and enlarged vessel radii. These findings challenge the assumption that HAs dominate HCC blood flow, potentially explaining the limited efficacy of TACE in diffuse HCC cases. This study provides high-resolution anatomical insights into liver vasculature, offering a foundation for improved tumor-targeting therapies.

## 1. Introduction

Liver diseases, such as hepatocellular carcinoma (HCC), liver cirrhosis, liver failure, and chronic viral or non-viral hepatitis, have become one of the leading causes of death globally over the past several decades [1]. Liver cancer is the sixth most common cancer and the third leading cause of cancer-related mortality [2,3]. The liver is a highly vascularized organ containing a dense vascular system. Moreover, alterations in its vasculature play a significant role in the development of liver diseases. Although the etiology of liver tumors varies, including hepatic virus infection, alcohol use, and nonalcoholic fatty liver disease, hypervascularity and marked vascular abnormalities are the most common pathological features [4,5].

The complicated vascular network of the liver comprises portal veins (PVs), hepatic veins (HVs), and hepatic arteries (HAs). These vessels are connected by hepatic sinusoids, and the liver mainly receives blood from the PV, which delivers nutrients and toxins from peripheral organs. The PV is responsible for approximately 70% of the blood flow into the liver. However, in contrast to the dual vascularization (HA and PV) of the liver parenchyma, HCC lesions are considered to have a vascular supply that is primarily dependent on the HA. Based on this feature, transarterial chemoembolization (TACE) has been developed and is recommended as the standard care for patients with intermediate- and advanced-stage HCC. However, its efficacy remains unsatisfactory. TACE-refractory and high tumor recurrence rates after the procedure are urgent problems [6]. Therefore, there is a need to further investigate the microvascular changes in liver tumors and abandon the one-size-fits-all strategy [7].

Although drastic improvements have been achieved in vascular imaging, a cutting-edge investigation technique is still required to understand the microvascular architecture of the liver. Currently, immunofluorescence and micro-computed tomography (CT) are the most commonly used methods for studying hepatic vasculature characteristics in research. However, several technical issues, including low resolution, perfusion difficulties, and transparency, accompany micro-CT [8,9]. Immunofluorescence is labor-intensive and challenging for large specimens, often causing information loss and structural distortion during serial sectioning. Moreover, traditional research methods cannot simultaneously acquire blood vascular structures, both at the organ level and at single-cell resolution [10,11]. The high-definition fluorescence-micro-optical sectioning tomography (HD-fMOST) technique enables breakthrough high-resolution whole-organ imaging through simultaneous imaging and sectioning. It has been successfully used to provide the first mesoscopic description of the entire vasculature of the mouse brain and lung [11,12,13,14,15]. Zhang et al. established HD-fMOST for reconstructing portal veins, hepatic arteries, bile ducts, and sinusoids in murine liver at single-cell resolution, creating the first 3D atlas of normal liver vasculature. However, pathological remodeling in HCC at this scale remains unclear [16].

Here, we established a pipeline of hepatic microvascular imaging through HD-fMOST to provide high-resolution quantitative anatomical data on the characteristics and architecture of liver vasculature in wild-type (WT) mice and Akt/Ras HCC mouse models, which has significant implications for the further development of new tumor vasculature-targeting therapies.

## 2. Materials and Methods

### 2.1. Animal Sample Preparation

This study involved the use of two male WT C57BL/6 mice and an Akt/Ras HCC mouse. The mice were obtained from Shouzheng Pharma Biotechnology Co., Ltd. (Wuhan, China). All mice were housed in well-ventilated cages on a 12 h light/dark cycle, and the temperature and the humidity were controlled at 22–26 °C and 40–70%, respectively. The mice had ad libitum access to food and water.

The establishment of the Akt/Ras HCC mouse model has been described in previous studies [17,18]. Exactly 2 μg of Sleeping Beauty transposon, 25 μg of NRAS (NRASG12V), and 25 μg of myristoylated Akt plasmids were diluted in physiological saline (0.9% solution of sodium chloride in a volume equivalent to 10% of body weight), filtered through a 0.22 μm filter, and injected into the lateral tail vein of 6-week-old C57BL/6 mice within 5 to 7 s. Five weeks after the injection, all mice underwent perfusion. The mice were anesthetized with 1% sodium pentobarbital (Sigma, St. Louis, MO, USA) (60 mg/kg) and subsequently, 0.01 M of phosphate-buffered saline (PBS) (Sigma-Aldrich Inc., St. Louis, MO, USA) was perfused into their hearts, followed by the addition of 4% of paraformaldehyde (Sigma-Aldrich) and 0.03% (*w*/*v*) of gelatin and fluorescein isothiocyanate (FITC) at 40 °C. Then, the right anterior lobes of the liver were dissected and fixed in 4% paraformaldehyde at 4 °C for 24 h. They were then rinsed thrice in 0.01 M of PBS at 4 °C. It took 6 h each for the first two washes and 12 h for the third wash. The liver tissues were dehydrated using a graded series of ethanol solutions (50, 70, and 95%), and 100% ethanol was used for further dehydration for 2 h (all at 4 °C). Subsequently, the tissues were sequentially immersed in LR White Resin (R1280A, uncatalyzed; Agar Scientific Ltd., Rotherham, UK). All animal experiments followed the procedures approved by the Fourth Military Medical University and were reported in accordance with ARRIVE guidelines.

### 2.2. Imaging

The HD-fMOST system was used to perform dual-wavelength imaging of the mice’s liver tissues. The green channel acquired the fluorescent signals of the liver vasculature, and the red channel acquired propidium iodide (PI)-labeled cytoarchitecture information. Using HD-fMOST, we simultaneously obtained vessel structures and cytoarchitectural information in an intact liver lobe at a single-cell resolution. The HD-fMOST system performed dual-wavelength imaging with the green channel at 475 nm for FITC-labeled vasculature and the red channel at 561 nm for PI-stained cytoarchitecture. The dataset acquired from the right anterior lobe of WT mouse contained 2713 coronal planes with a data size of 56.1 TB, whereas the dataset from the right anterior lobe of HCC mouse contained 4548 coronal planes with a data size of 162 TB.

### 2.3. Image Preprocessing and Vessel Reconstruction

To reconstruct large-caliber vessels in the liver, a median filter was applied to the original resolution datasets for denoising, followed by gamma correction to enhance image contrast. The optical resolution was 0.35 × 0.35 × 2 μm. Multiple cycles of automatic sectioning were performed with an axial step size of 2 μm. Imaris software (10.0.0, Oxford Instruments, Abingdon, UK) was used to skeletonize the vessels within and distinguish the HAs, HVs, and PVs. Manual tracing and skeletonization of the sinusoids were also performed using Imaris software.

### 2.4. Histology and Immunohistochemistry

Hematoxylin and eosin (H&E), Masson’s trichrome, Ki67, and lymphatic endothelial receptor-1 (LYVE1; a marker of liver sinusoid endothelial cells) stainings were performed on the harvested liver tissues of the WT and Akt/Ras HCC mice. Liver tissues were fixed in 10% phosphate-buffered formalin for a period of at least 24 h, processed using routine histology procedures, embedded in paraffin, sectioned into 4 μm pieces, and mounted on a slide. Thereafter, the sections were stained with H&E dye and Masson’s trichrome staining reagents, following standard steps, to evaluate the severity of the pathological changes. Hepatocyte proliferation and sinusoid capillarization were quantified using Ki-67 and LYVE1 immunofluorescence staining, respectively. Anti-Ki67 (1:1000, GB121141; Servicebio, Wuhan, China) and anti-LYVE1 (1:2000, GB113499; Servicebio) antibodies were used for immunofluorescence staining. The proportion of positive cells (Ki67%) and LYVE1-positive area was quantified using Image J software (version 1.54g). Images were acquired using a NIKON fluorescence microscope (ECLIPSE C1, Nikon Corporation, Tokyo, Japan).

### 2.5. Statistical Data Analysis

Nonparametric Kruskal–Wallis test was conducted, with statistical significance indicated at *p* < 0.01–0.001. All statistical analyses were performed using the SPSS software (version 17.0; IBM Corp, New York, NY, USA).

## 3. Results

### 3.1. Validation of the Gelatin-FTIC Perfusion Method for Liver Vasculature Acquisition

**Figure 1:**
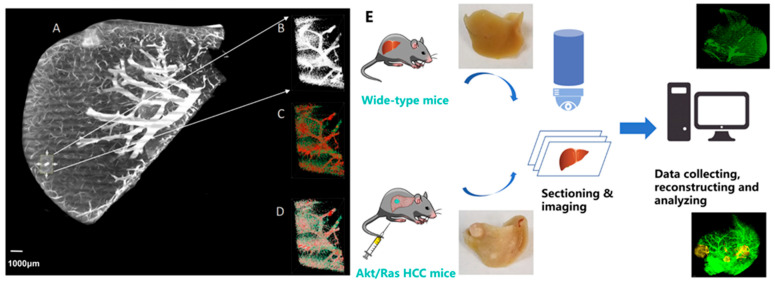
Schematic illustration of the strategy for reconstructing the liver vascular architectures. (**A**) Overall illustration of the normal mouse liver; (**B**) partial data block illustration (the size of the data block is X800 × Y1100 × Z700 μm); (**C**) effect illustration of the partial data block after skeletonization (the skeletonization operation can determine the centerline of the blood vessel, which is convenient for subsequent statistical analysis of blood vessel length, density, and volume fraction); and (**D**) illustration of the combination of (**B**,**C**). (**E**) Procedure for acquiring datasets using high-definition fluorescence micro-optical sectioning tomography (HD-fMOST), in which imaging and sectioning were performed simultaneously. Scale bar, 1000 μm: (**A**).

First, we established a pipeline for hepatic vascular system acquisition using gelatin and FITC perfusion with the HD-fMOST system. Figure 1A illustrates the coronal image of a normal liver after preprocessing, which was visualized from 4407 subgraphs. Multiple cycles of automatic sectioning were performed with an axial step size of 2 μm. In the selected liver tissue (X800 × Y1100 × Z700 μm), the total vascular length, volume, and mean radius were 1,336,188.5 μm, 27,048,346 μm^3^, and 0.56620502 μm, respectively. The skeletonization operation determined the centerline of the blood vessels (Figure 1B–D). The procedure for acquiring an image dataset using HD-fMOST is illustrated in Figure 1E. The mouse liver was segmented into five lobes: the left lateral, median, right anterior, right posterior, and caudate lobes. We reconstructed the vascular systems of the right anterior lobe in both the HCC and normal liver models. The sectioned volumes of normal liver and HCC tissues were 204.8 mm^3^ and 212.8 mm^3^, respectively.

### 3.2. Histopathology Analysis

**Figure 2:**
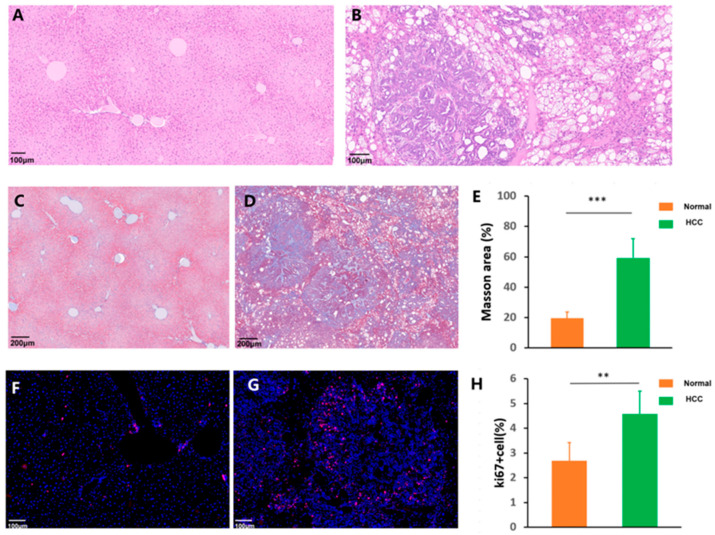

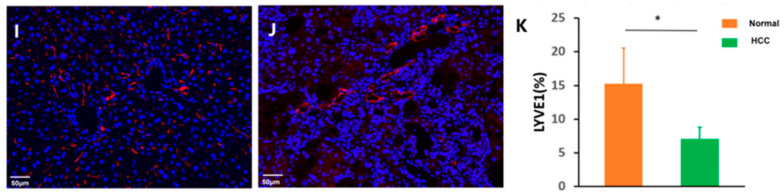
Histopathological changes between normal and Akt/Ras hepatocellular carcinoma (HCC) mice. Microscopic images (×20) of the liver sections following hematoxylin and eosin (H&E) staining: (**A**) a normal hepatic architectural pattern and (**B**) the Akt/Ras HCC mouse exhibited marked fatty degeneration with vacuolated cytoplasm, hyperchromasia, and abnormal mitotic figures with pleomorphic nuclei. Masson’s trichrome staining for collagen deposition: (**C**) normal mouse liver; (**D**) Akt/Ras HCC mouse liver; and (**E**) positive area following Masson’s trichrome staining analyzed using ImageJ. Ki67 immunofluorescence staining: (**F**) normal mouse liver; (**G**) Akt/Ras HCC mouse liver; and (**H**) Ki67 positive cell percentage analyzed using ImageJ. LYVE1 immunofluorescence staining: (**I**) normal mouse liver; (**J**) Akt/Ras HCC mouse liver; and (**K**) LYVE1-positive area analyzed using ImageJ. Scale bar, 100 μm: (**A**,**B**,**F**,**G**); 200 μm: (**C**,**D**). 50 μm: (**I**,**J**); N = 5 sections/group. * *p* < 0.05, ** *p* < 0.01, and *** *p* < 0.001.

The pathological examination of H&E-stained liver sections from a control normal mouse showed a normal hepatic architectural pattern (Figure 2A). Normal liver tissues displayed an intact lobular structure with clear central veins and radiating hepatic cords without necrosis and inflammation. However, the Akt/Ras mouse exhibited multinodular diffuse HCC cells, and malignant neoplasms had replaced most of the normal liver tissues. H&E staining showed a loss of liver architecture and typical pathological characteristics, including marked fatty degeneration with vacuolated cytoplasm, obvious collagen depositions, nuclear enlargements, hyperchromasia, and abnormal mitotic figures with pleomorphic nuclei, demonstrating the establishment of the HCC model (Figure 2B). Pathological features of fibrosis were identified using Masson’s trichrome staining. Liver sections from the Akt/Ras mouse showed remarkable liver fibrosis compared to normal livers (Figure 2C–E). Ki67 immunofluorescence staining was performed to determine the number of proliferating hepatocytes. The Akt/Ras HCC mouse exhibited an increased number of Ki67-positive cells (Figure 2F–H). LYVE1 reflects capillarization characteristics [19]. The results of the LYVE1 immunofluorescence staining showed that LYVE1-stained cells were significantly decreased in the HCC model (Figure 2I–K).

### 3.3. Three-Dimensional Morphological Features of the Microvascular Systems

**Figure 3:**
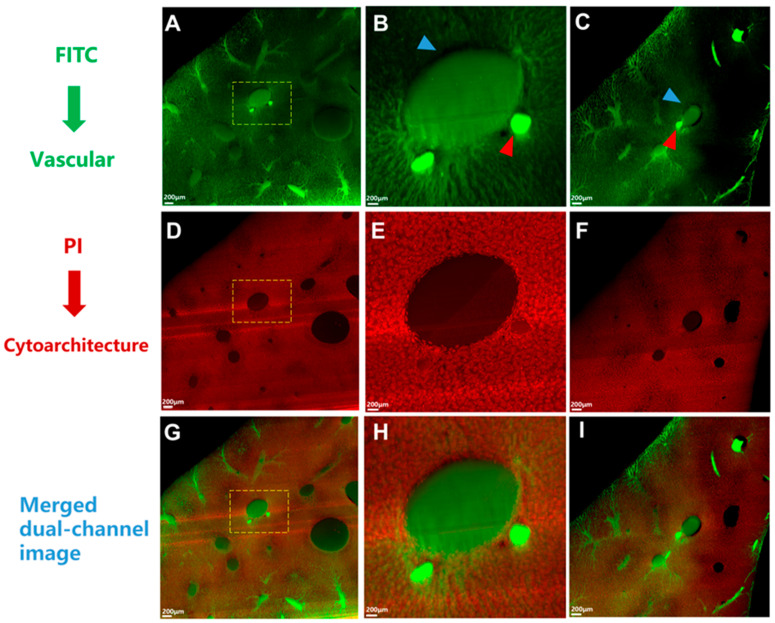
Dataset acquired by high-definition fluorescence micro-optical sectioning tomography (HD-fMOST) comprising two image channels containing vascular and cytoarchitecture information. (**A**–**C**) The green channel indicated the vessel channel. (**D**–**F**) The red channel indicated the cytoarchitecture channel. (**G**–**I**) The merged two-channel image of the same location of a liver tissue section. The portal vein is marked with a blue arrowhead, and the hepatic artery is marked with a red arrowhead. Scale bar, 200 μm.

The dataset acquired by HD-fMOST comprised two image channels containing vascular and cytoarchitecture information with a resolution of 0.35 × 0.35 × 2 μm. The green channel indicated the vessel channel (Figure 3A–C). The red channel indicated the cytoarchitecture channel (Figure 3D–F). Figure 3G–I illustrates a merged two-channel image of the same location in a liver tissue section. The HA, with a relatively stable lumen and regular shape, was accompanied by the PV. We distinguished between the PV and HV according to the principle that the PV was accompanied by the HA, while the HV was not accompanied by other vessels [16].

**Figure 4:**
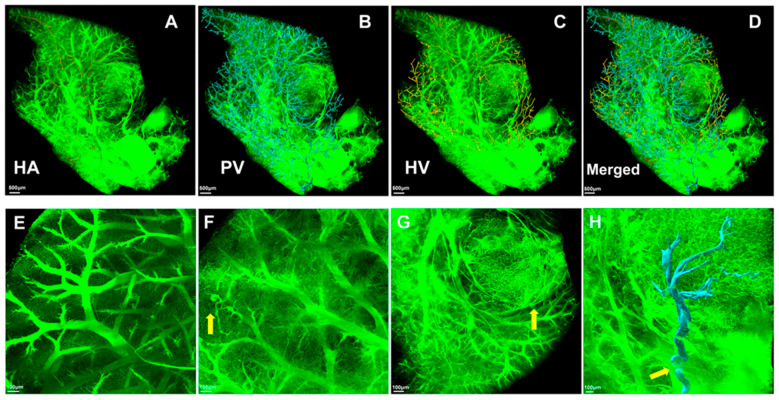
Microvascular mapping by HD-fMOST. (**A**) HA (red); (**B**) PV (blue); (**C**) HV (yellow); (**D**) merged images; (**E**) illustration of the microvascular morphology of normal liver tissues; (**F**) the vasculature was twisted and disordered (yellow arrow); (**G**) the vasculature was compressed by tumor nodules (yellow arrow); and (**H**) 3D reconstruction of the tortuous main PV (blue) that supplied the tumor with sudden sharp bends (yellow arrows). Scale bar, 500 μm: (**A**–**D**); 100 μm: (**E**,**F**,**H**). 30 μm: (**G**).

The HA (red), PV (blue), HV (yellow), and merged images are illustrated in Figure 4A–D, respectively. The PV and HV were similarly and parallelly distributed throughout the liver lobe in a fan shape. Regarding the microvascular morphology, compared with normal liver tissues, we found that the flow of blood vessels became highly irregular in the tumor tissues (Figure 4E–G). Moreover, the microvascular systems were twisted, disordered, and compressed by the tumor nodules (yellow arrow). Figure 4H illustrates a 3D reconstruction of the main PV (blue) that supplied the tumor. It was significantly affected by mechanical compression and became more tortuous, resulting in sudden sharp bends (yellow arrows).

**Figure 5:**
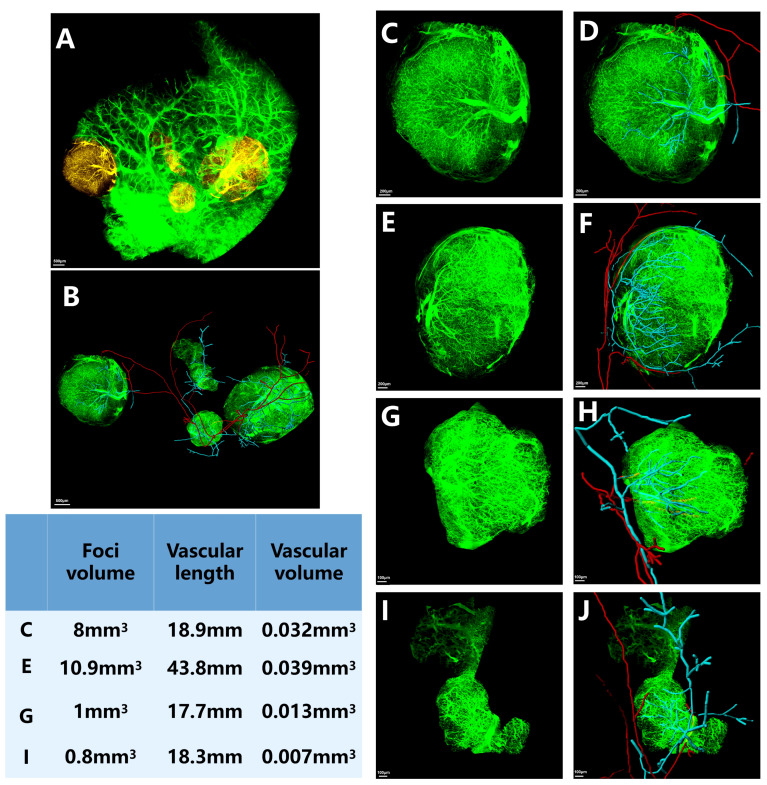
Microvasculature in the four tumor lesions observed using HD-fMOST. (**A**) The area of the tumor lesions is indicated in yellow; (**B**) illustration of the vessels supplying the tumors after removal of the liver background; and (**C**–**J**) zoom-in of the four tumor lesions and their inflow vessels. The HA and PV are shown in red and blue, respectively. Scale bar, 500 μm: (**A**,**B**); 200 μm: (**C**–**F**). 10 μm: (**G**–**J**).

Four tumor lesions were observed in the right anterior lobe of the HCC model (Figure 5). The tumor lesion volumes were 8.0 mm^3^, 10.9 mm^3^, 1.0 mm^3^, and 0.8 mm^3^, respectively. Our findings revealed that the HA was not the primary source of tumor blood supply in these multinodular lesions. Instead, the PV served as the dominant source of tumor blood inflow, with its vascular branches occupying a more central position within the tumors. The length of PVs was consistently greater than HAs (*p* = 0.021). Notably, the HA typically coursed along the tumor periphery rather than penetrating the core. The PV emerged as the principal vascular supply for the tumors, with detailed vascular parameters provided in Supplementary Table S1.

**Figure 6:**
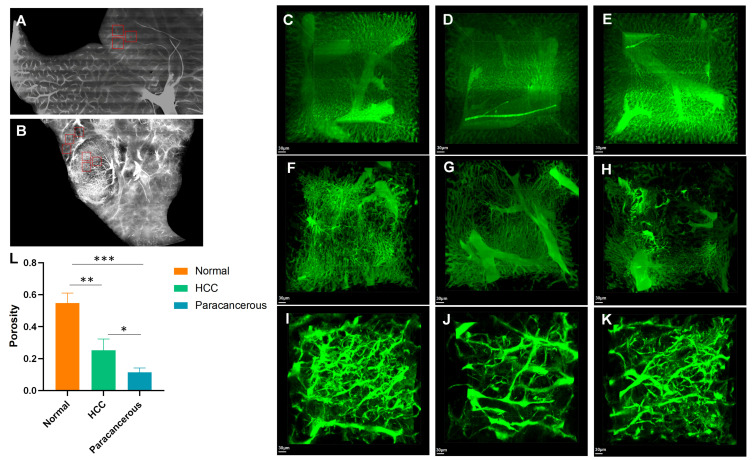
Porosity analysis of normal tissues, paratumoral cirrhotic tissues, and tumor tissues. (**A**) Three blocks were randomly chosen from the normal liver tissues. (**B**) Three blocks were randomly chosen in the paratumoral cirrhotic (left) and tumor tissues (right). (**C**–**E**) Zoom-in of the blocks indicated in the red boxes (**A**); (**F**–**H**) zoom-in of the blocks indicated in the left red boxes (**B**); (**I**–**K**) zoom-in of the blocks indicated in the right red boxes (**B**). (**L**) Comparison of porosity among normal tissues, paratumoral cirrhotic tissues, and tumor tissues. * *p* < 0.05, ** *p* < 0.01, and *** *p* < 0.001. Scale bar, 30 μm: (**C**–**K**).

In the vessel channel, we selected three blocks randomly to represent normal tissues, paratumoral cirrhotic tissues, and tumor tissues with a size of 400 × 400 × 400 μm^3^ in the three groups (Figure 6A,B). Porosity refers to the volume of blood vessels per unit volume. We found that the porosity of normal liver tissues (Figure 6C–E) was significantly greater than that of HCC (*p* = 0.005, Figure 6F–H) and paracancerous cirrhotic tissues (*p* < 0.001, Figure 6I–L).

### 3.4. Cirrhosis and Tumors Specifically Affected the Sinusoidal Network Zone

**Figure 7:**
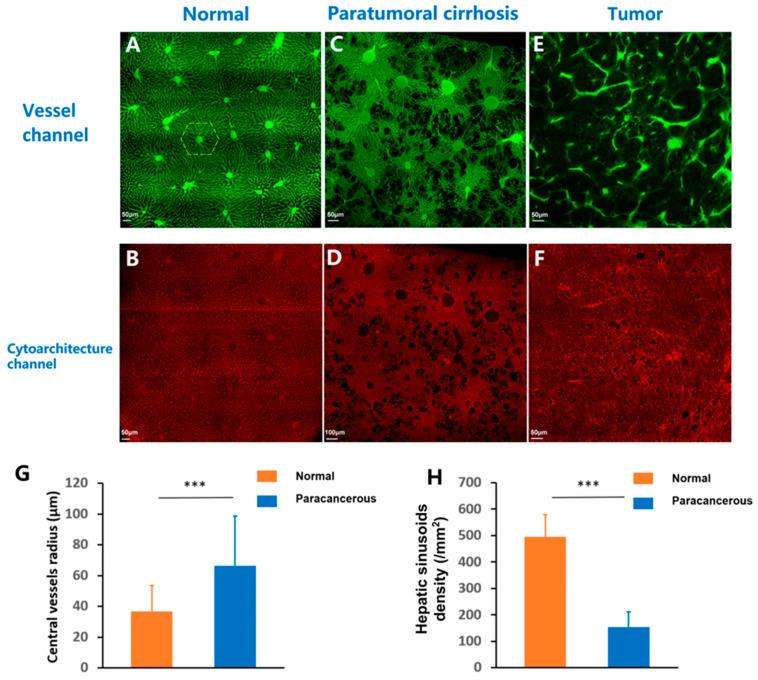
Illustration of the sinusoidal network zone observed using HD-fMOST. (**A**) Structures of the liver sinusoids from the vascular channel in the normal liver tissues. A typical normal hepatic sinusoidal structure is marked using a yellow line. (**B**) Cytoarchitecture channel of the same section as in (**A**) following PI staining. (**C**) Structures of the liver sinusoids from the vascular channel in the paratumoral cirrhotic tissues. (**D**) Cytoarchitecture channel of the same section as in (**C**) following PI staining. (**E**) Structures of the liver sinusoids from the vascular channel in the tumor tissues. (**F**) Cytoarchitecture channel of the same section as in (**E**) following PI staining. (**G**) The radii of the central vessels in the hepatic sinusoids of the normal tissues were significantly smaller than those of paratumoral cirrhotic tissues. (**H**) The hepatic sinusoid density of the normal tissues was larger than that of the paratumoral cirrhotic tissues. *** *p* < 0.001. Scale bar, 50 μm: (**A**,**B**,**E**,**F**); 100 μm: (**C**,**D**).

Aside from being significantly different from the normal tissues, the structures of the liver sinusoids in paratumoral cirrhotic and tumor tissues were significantly destroyed, and the regular structure disappeared (Figure 7). A typical normal hepatic sinusoidal structure was marked by a yellow dashed line, which was regular and recognizable in the normal liver tissues (Figure 7A,B). However, it was difficult to recognize the hepatic sinusoids in the paratumoral cirrhotic tissue, and the sinusoids completely disappeared in the tumor tissues (Figure 7C–F). The radii of the central vessels in the hepatic sinusoids of the normal tissues were significantly less than those of the paratumoral cirrhotic tissues (36.8 vs. 66.4 μm, *p* < 0.001) (Figure 7G). However, the hepatic sinusoid density of the normal tissues was greater than that of the paratumoral cirrhotic tissues (496 vs. 154.3 μm^−2^, *p* < 0.001) (Figure 7H).

## 4. Discussion

Using the state-of-the-art HD-fMOST technique, we illustrated the arrangement of liver vascular systems in both normal and HCC model mice at micrometer resolutions up to the order of 0.35 μm. We assessed and quantified the intricate pathological vascular networks of liver tumors. The results showed that the vascular-supplying tumors in the mouse model were enlarged and tortuous. Liver sinusoidal remodeling was abundant, and the diameter and number of sinusoids significantly decreased. The data demonstrated various anatomical vascular abnormalities attributable to liver tumors.

Unlike other visceral organs, the liver has intricate vascular networks. Angiogenesis plays an essential role in the development of liver tumors, and it is valuable for research [4]. In contrast to the dual vascularization (HA and PV) of the liver parenchyma, HCC cells are considered to have a vascular supply that is mostly dependent on the HA. In the TACE procedure, chemotherapy and embolization agents are delivered to the tumor through the HA [20]. However, TACE has been reported to be more effective for the treatment of nodular HCC than for diffuse/infiltrative HCC. Multinodular or infiltrative HCC is considered a condition for which TACE is unsuitable, and its underlying mechanism remains unclear.

The preclinical animal model used in this study was of the diffuse type, meaning numerous, discrete, small tumor nodules were scattered throughout the liver. We examined the circulation characteristics of the tumor systems at the micro level. Tumor tissue growth exerts a compressive force on the surrounding blood vessels. This compression narrows the pliant vascular system, which contributes to increased portal pressure. Compared to normal liver tissues, the radii of the central vessels in the hepatic sinusoids in paratumoral cirrhotic tissues were significantly increased; however, the hepatic sinusoid density was decreased. Our study demonstrated that liver fibrosis not only induces a sinusoidal density decrease but also promotes a sinusoidal arrangement with dilated central vessels. In tumor tissues, all hepatic sinusoids disappeared, and all microvascular structures displayed loops. Most importantly, in the four microtumor lesions, we found that the HA was not the main tumor blood inflow vessel, and its path was at the tumor margin. Instead, the number of PVs was significantly higher than that of the HAs, and the PV constituted the main source of tumor blood inflow. The PV occupied a central position and constituted the main blood vessel that supplied the tumors. This may explain the poor efficacy of TACE in patients with diffuse/infiltrative HCC. The standard TACE beads (100–300 μm) used clinically primarily target hepatic arterioles [21]. However, our HD-fMOST analysis demonstrates that tumor-supplying portal venules have significantly larger diameters (80–150 μm). This vascular caliber mismatch may underlie the limited treatment efficacy in portal-venule dominant HCC subtypes.

In addition, in the current study, we established a gelatin-FITC fluorescent glue vascular perfusion method that could stably obtain 3D volume data with a high signal-to-noise ratio and facilitate the visualization of the liver vascular network. Gelatin is a colorless and tasteless natural macromolecule derived from collagen and is widely used as a gel in food and medicine [22]. Gelatin has low viscosity in the liquid state and can easily fill the blood vessels of the entire body through perfusion. After low-temperature solidification, it can undergo routine procedures, such as slicing. Moreover, it is compatible with 4% paraformaldehyde and is suitable for vascular network visualization research. Beyond vascular visualization, the liver decellularization model has broad applications in biomedical research and drug development [23]. However, the distinctive feature of the fMOST technique lies in its ability to perform continuous liver tissue slicing with an axial step size of 2 μm, simultaneously capture images, and provide real-time counterstaining using PI. As a result, HD-fMOST bypasses the need for extracellular matrix removal, thereby avoiding potential shrinkage artifacts and reducing time-consuming [24,25].

Fueled by the development of imaging technology, methods for liver vessel imaging have significantly improved. CT and magnetic resonance imaging can reveal the liver vascular structure at resolutions of 0.5–2 mm. The emergence of micro-CT has particularly enhanced the imaging resolution of liver vessels in scientific research [26,27]. Debbaut et al. used casting resin mixed with color dyes to perfuse a normal human liver after hepatectomy to visually achieve hepatic vasculature and 3D reconstruction [28]. However, it was suggested that casting may lead to slight shrinkage of the resin. Moreover, during that time, it was technically impossible to scan the tissue or analyze an image file with an extremely large size at a resolution of micrometers because of computational issues. Therefore, liver samples with smaller sample sizes were scanned. At the macrocirculation level, the size of the scanned sample was approximately 88 mm^3^ at a resolution of 71 μm. At the microcirculation level, the size of the scanned sample was 2 mm^3^ at a resolution of 2.6 μm. In 2017, Peeters et al. made some technical improvements. They combined immunohistochemistry technology with micro-CT to increase the visualization depth and reconstructed the hepatic circulation in a rat model [29]. However, the sample size was still relatively small (2.5 × 2.5 × 2.5 mm^3^), and the slice for IHC was as thick as 350 μm. Their study confirmed that the HA and PV lay parallel to each other, which is similar to that in our study. Later, they further analyzed the 3D distribution of blood vessels in a thioacetamide-induced liver cirrhosis rat model using the same technique and found that liver cirrhosis induced vessel collapse or sudden sharp bend formations [8]. The micro-CT technique enables visualization of intact organs in 3D without physical sectioning, but its limitation lies in resolution constraints, which prevent detailed differentiation of microstructures like liver lobules [26]. Compared to that in previous studies, we scanned the whole right anterior lobe and reconstructed the vascular systems with a larger sample size and a volume of about 200 mm^3^ at an extremely high resolution (2 μm). In addition, current clinical imaging modalities, including ultrasound, MRI, and CT, have difficulty distinguishing the hepatic lesions from cirrhotic background parenchyma, particularly demonstrating low sensitivity for early detection of microvascular abnormalities. In contrast, our technique directly visualizes pathological changes such as microvascular distortion and compression, overcoming the resolution limitations of conventional imaging for micron-scale structures [30].

This study had several limitations. First, the number of animal studies was limited because of high testing costs. Second, we characterized several anatomical variants; however, more variants certainly exist. The information obtained from PI staining needs to be thoroughly explored in future studies. It would be valuable to validate the significance of 3D imaging in the diagnosis of complex hepatic pathologies. Third, we hypothesized that the poor efficacy of TACE for diffuse/infiltrative HCC might be because the PV constitutes the main inflow of tumor blood vessels. While our findings provide detailed insights into the microvascular architecture of this subtype, we acknowledge that other HCC subtypes (e.g., nodular or heterogeneous forms) may exhibit different vascular patterns. Further comparative studies across additional HCC models or subtypes would be valuable to validate the generalizability of our results.

## 5. Conclusions

Our study provides a deeper understanding and an advanced research method for normal liver microvasculature and alterations in cases of cirrhosis and HCC, which is an essential next step for further studies in the fields of hepatology and liver tumors. The novel data gathered in this study complements the scientific insights into liver morphology and physiology. This straightforward and novel technical approach can be used in multiscale clinical pathology and preclinical animal studies. Future studies using this 3D liver microvascular approach should combine it with multi-omics to enhance our understanding of the physiological and pathophysiological aspects of liver diseases.

## Data Availability

The data generated during the current study are available from the corresponding author upon reasonable request.

## Supplementary Materials

The following supporting information can be downloaded at: https://www.mdpi.com/article/10.3390/cancers17162653/s1, Supplementary Table S1. Quantitative analysis of vascular parameters in four representative lesions (corresponding to Figure 5).

## Abbreviations

The following abbreviations are used in this manuscript:

CT	computed tomography
FITC	fluorescein isothiocyanate
H&E	hematoxylin and eosin
HA	hepatic artery
HCC	hepatocellular carcinoma
HD-fMOST	high-definition fluorescence micro-optical sectioning tomography
HV	hepatic vein
LYVE1	lymphatic endothelial receptor-1
PBS	phosphate-buffered saline
PI	propidium iodide
PV	portal vein
TACE	transarterial chemoembolization
WT	wild-type
